# Surgical Site Infection after Breast Surgery: A Retrospective Analysis of 5-Year Postoperative Data from a Single Center in Poland

**DOI:** 10.3390/medicina55090512

**Published:** 2019-08-21

**Authors:** Anna Palubicka, Radoslaw Jaworski, Marcin Wekwejt, Beata Swieczko-Zurek, Michal Pikula, Janusz Jaskiewicz, Jacek Zielinski

**Affiliations:** 1 Department of Laboratory Diagnostics and Microbiology with Blood Bank, Specialist Hospital in Koscierzyna, 83-400 Koscierzyna, Poland; 2 Department of Surgical Oncology, Medical University of Gdansk, 80-210 Gdansk, Poland; 3 Department of Cardiac Surgery, Children’s Health Memorial Institute, 04-730 Warsaw, Poland; 4 Faculty of Mechanical Engineering, Department of Materials Engineering and Bonding, Biomaterials Group, Gdansk University of Technology, 80-233 Gdansk, Poland; 5 Department of Clinical Immunology and Transplantology, Medical University of Gdansk, 80-210 Gdansk, Poland

**Keywords:** surgical site infection, breast surgery, breast implants, complications

## Abstract

*Background and Objectives:* Surgical site infection (SSI) is a significant complication of non-reconstructive and reconstructive breast surgery. This study aimed to assess SSI after breast surgery over five years in a single center in Poland. The microorganisms responsible for SSI and their antibiotic susceptibilities were determined. *Materials and Methods:* Data from 2129 patients acquired over five years postoperatively by the Department of Surgical Oncology, Medical University of Gdansk in Poland were analyzed. *Results:* SSI was diagnosed in 132 patients (6.2%) and was an early infection in most cases (65.2%). The incidence of SSI was highest in patients who underwent subcutaneous amputation with simultaneous reconstruction using an artificial prosthesis (14.6%), and breast reconstruction via the transverse rectus abdominis muscle (TRAM) flap method (14.3%). Gram-positive bacteria were responsible for SSI in most cases (72.1%), and these were mainly *Staphylococcus* strains (53.6%). These strains were 100% susceptible to all beta-lactam antibiotics (except penicillin) but were less susceptible to macrolides and lincosamides. *Conclusions:* SSI is a serious problem, and attention should be focused on its prevention. Reconstruction using an artificial prosthesis or via the TRAM flap method is connected to increased SSI incidence. Further studies are required to prevent SSI following breast surgery.

## 1. Introduction

Surgical site infection (SSI) is one of the most common and serious complications following surgery. The occurrence of SSI varies according to the type of operation, wound cleanliness, and the operative field. This complication can lead to prolonged hospitalization, which increases the cost of treatment [1,2,3]. SSI occurs frequently following breast surgery [4] because such surgery is mainly performed to treat breast cancer, and consequently the tissue is subjected to chemotherapy and/or radiotherapy. Current surgical options include breast-saving techniques, mastectomy, autograft techniques, use of an acellular dermal matrix, implantation of breast implants, and a combination of these methods [4,5,6,7]. The risk of complications, including SSI, is affected by the type of breast surgery and whether breast reconstruction is performed. A variety of risk factors for SSI following breast cancer surgery have been reported, including older age, obesity, alcohol abuse, smoking, diabetes, malignancy, previous open biopsy, breast-conservation surgery, previous radiation therapy or chemotherapy, surgeon experience, seroma development, prolonged duration of drainage, immediate reconstruction, and lack of antibiotic prophylaxis at the time of surgery [8]. Although the majority of breast procedures are considered clean operations, the SSI rates reported in the individual studies remain higher than would be expected. Olsen et al. reported that SSI rates in mastectomy without immediate reconstruction, mastectomy with implant reconstruction, and mastectomy with autologous flap reconstruction were approximately 3%–18%, 0.4%–17%, and 1%–12%, respectively [8]. Staphylococci are the organisms isolated most commonly in SSI after breast surgery (60%), whereas Gram-negative bacilli and anaerobes account for 40% [9]. Susceptibility testing of the staphylococcal isolates found drug resistance in 63% [9]. In patients with breast implant infection, the vast majority of isolates are Gram-positive microorganisms (83%), with the rate of methicillin-sensitive staphylococci of 49% and a much lower proportion of infections due to methicillin-resistant vs. susceptible *Staphylococcus aureus* (MRSA vs. MSSA, 3.5% vs. 30.6%, respectively) [10].

This study aimed to assess SSI after breast surgery over five years in a single center in Poland. The microorganisms responsible for SSI and their antibiotic susceptibility were determined.

## 2. Methods

This cohort study used the medical records of patients who underwent breast surgery due to neoplasms at the Department of Surgical Oncology, Medical University of Gdansk in Poland between 2012 and 2016. The study population included 2129 patients, and was divided into those who underwent classic breast surgery (without breast-saving techniques), breast-conserving surgery, breast reconstruction via the transverse rectus abdominis muscle (TRAM) flap method, and subcutaneous amputation with simultaneous reconstruction using an artificial prosthesis.

SSI was defined as any episode of clinical symptoms of infection following surgery, or when SSI was diagnosed by the surgeon. Antibiotic use and surgical removal of an implant were at the surgeon’s discretion. Early and late SSI were defined by whether the onset of symptoms occurred within 30 days or more than 30 days after surgery, respectively.

The age, body mass index, hospitalization duration, smoker status, and comorbidities (diabetes and hypertension) of patients diagnosed with SSI were assessed. Samples were acquired from these patients for microbiological evaluation. The microorganisms responsible for SSI were determined, and their antibiotic susceptibility was assessed after excluding samples that were contaminated and affected by pre-laboratory and human errors.

Continuous data are presented as means or medians, standard deviations (SDs), and ranges. Categorical data are presented as percentages. The Kolmogorov–Smirnov test was used to test whether the data were normally distributed. Continuous data were compared using a univariate analysis of variance followed by the least significant difference test or the Kruskal–Wallis test, depending on their distribution. Categorical data were compared using the chi-square test and Fisher’s exact test. *P*-values less than 0.05 were considered statistically significant. All statistical analyses were performed using SPSS software v.21 (IBM, Chicago, IL, USA).

This study was approved by the Ethics Examining Committee of Human Research at Medical University of Gdansk in Poland (approval no. 424/2017, date of approval: 27 November 2018).

## 3. Results

A total of 2129 breast surgical interventions were conducted at our center in 2012–2016, with a median of 408 procedures per year (SD = 55.7; range 502–358). Classic breast surgery, breast-conserving surgery, breast reconstruction via the TRAM flap method, and subcutaneous amputation with simultaneous reconstruction using an artificial prosthesis were performed in 40%, 47%, 3%, and 10% of the sampled patients, respectively.

Over five years, 132 patients (6.2%) were postoperatively diagnosed with SSI. These were early infections in most cases (65.2%). The number of patients who developed SSI was similar each year; a mean of 26 patients (6.4%) were diagnosed with SSI annually (SD = 10.2; range 15–41). Table 1 shows the number of patients in each treatment group who were diagnosed with SSI. The incidence of SSI was significantly higher in patients who underwent subcutaneous amputation with simultaneous reconstruction using an artificial prosthesis (14.6%) and breast reconstruction via the TRAM flap method (14.3%) than in the other two groups. Patients with SSI had a median age of 55 years (SD = 13.1 years; range 34.1–74.6 years), a median body mass index of 26.1 (SD = 5.3; range 20.5–34.8), and a median hospitalization duration of five days (SD = 4.8 days; range 21–33 days). In total, 24 (18.2%) and 85 (64.4%) patients with SSI were smokers and had comorbidities, respectively.

**Figure 1:**
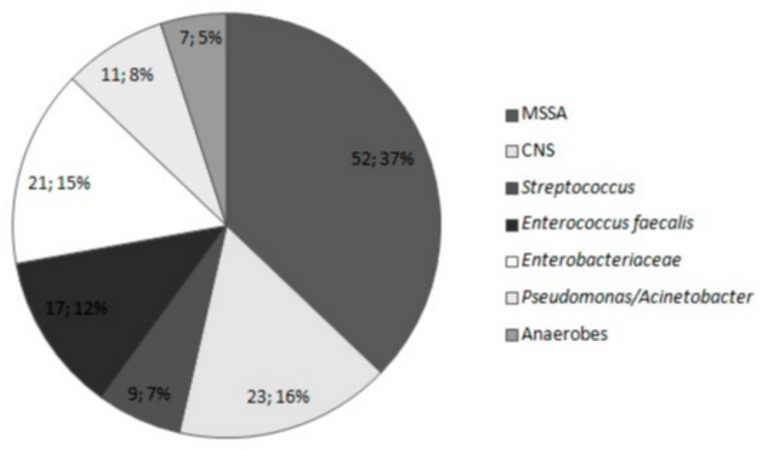
Microorganisms responsible for surgical site infection (SSI) in patients who underwent breast surgery (number; percentages). MSSA, methicillin-susceptible *Staphylococcus aureus*; CNS, coagulase-negative *Staphylococcus*.

Microbiological evaluation of samples from patients diagnosed with SSI yielded 106 positive results (80.3%) and 26 negative results (19.7%). Among the negative results, three samples (2.3%) were contaminated by skin due to pre-laboratory errors. A total of 140 strains were detected in the 101 patients with microbiologically confirmed SSI. One type of bacteria was isolated in the majority (76) of patients, whereas two or more types of bacteria were isolated in 30 patients (28.3%). Specifically, two and three types of bacteria were isolated in 26 and 4 patients, respectively. Gram-positive bacteria were responsible for the majority of infections (101, 72.1%), and most of the isolates (75, 53.6%) were *Staphylococcus* strains. The microorganisms responsible for SSI are presented in Figure 1 and Table 2.

Table 3 shows the susceptibility of the isolated bacteria to antibiotics. *Staphylococcus aureus* strains, the most common etiological factor, were susceptible to all beta-lactam antibiotics (except penicillin) and exhibited 100% sensitivity to aminoglycosides, trimethoprim/sulfamethoxazole, linezolid, and vancomycin. Conversely, only 89% of these strains were susceptible to macrolides and lincosamides.

## 4. Discussion

This study investigated the occurrence of SSI in patients who underwent breast surgery in a single surgical oncology center in northern Poland. The incidence of SSI after breast surgery was 6.2%. Although the SSI was mostly classified as superficial, it resulted in increased morbidity and increased duration of hospitalization. Recent reviews of the database of the National Surgical Quality Improvement Program showed that 1.4%–3.2% of patients develop SSI after breast surgery [11,12,13]. However, other studies have reported that the rate of SSI is up to 36% following specific procedures, such as modified radical mastectomy [14,15]. In the current study, the incidence of SSI was higher in patients who underwent subcutaneous amputation with simultaneous reconstruction using an artificial prosthesis and breast reconstruction via the TRAM flap method than in patients who underwent classic breast surgery and breast-conserving surgery. This is consistent with previous reports stating that the rate of complications is highest in patients who undergo breast reconstruction, especially with their own tissues [16,17].

Numerous patient- and procedure-related factors affect the risk of SSI. Therefore, a bundle approach that targets multiple risk factors is required to reduce the risk of bacterial contamination and improve patient defenses. Guidelines for the prevention of SSI issued by the Centers for Disease Control and Prevention emphasize the importance of rigorous patient preparation, aseptic practice, and attention to detail during surgery. Perioperative antibiotic prophylaxis is important to limit infectious complications. Care should be taken to choose an appropriate antibiotic, deliver it at the optimal time interval prior to surgery, and limit its use after surgery. Suboptimal prophylactic antibiotic dosing is a potentially modifiable risk factor for SSI following breast surgery. Olsen et al. reported that the risk of SSI is increased in patients who undergo mastectomy and in patients who are fitted with an implant or a tissue expander during surgery [8]. These findings could be used to develop a specific risk stratification index that predicts the risk of SSI following breast surgery, and to devise infection prevention strategies tailored for breast surgery patients [8].

In the current study, *Staphylococcus* species were most frequently isolated from patients with SSI. This is consistent with the previous finding that *Staphylococcus* species were isolated in 60% of cultures from patients who developed SSI after breast surgery [9,14]. Surprisingly, multi-drug-resistant variants were rarely identified in the current study, and no methicillin-resistant *S. aureus* strains were isolated. A previous study reported that methicillin-resistant *S. aureus* is responsible for up to 9.8% of SSI cases after breast surgery [9]. In the current study, 28% of isolated bacteria were Gram-negative strains. In a recent report of patients who developed SSI after breast reconstruction using implants, Gram-positive strains, including *Staphylococcus epidermidis* and *S. aureus*, were most frequently isolated, while 16% of isolates were Gram-negative bacteria such as *Pseudomonas aeruginosa* [18]. Similarly, Darragh et al. reported that *S. epidermidis*, *P. aeruginosa*, *Enterobacter cloacae*, *S. aureus*, and *Escherichia coli* are most frequently isolated from patients who develop SSI after breast surgery [19].

The limitations of this study include its design as a retrospective study and the small numbers of patients with important risk factors and SSI. Consequently, we were unable to analyze potential risk factors for the development of SSI in breast cancer patients. A larger cohort study of breast cancer patients is required to investigate such risk factors in detail, including when neoadjuvant and adjuvant chemotherapy and radiotherapy are utilized.

## 5. Conclusions

The average incidence of SSI after breast surgery remains constant worldwide and is increased when reconstruction is performed during primary surgery. SSI is a serious problem, and attention should be focused on its prevention. New surgical techniques are being developed, and further studies are required to prevent SSI following breast surgery.

## Figures and Tables

**Table 1:** Surgical site infection in breast cancer patients who underwent breast surgery at the Department of Surgical Oncology, Medical University of Gdansk in Poland in 2012–2016.

	Classic Breast Surgery (*n* = 865)	Breast-Conserving Surgery (*n* = 1002)	Breast Reconstruction via the TRAM Flap Method (*n* = 56)	Subcutaneous Amputation with Simultaneous Reconstruction (*n* = 206)	*p* Value
All SSI	46 (5.3%)	48 (4.8%)	8 (14.3%)	30 (14.6%)	<0.001
Early SSI	34 (3.9%)	31 (3.1%)	4 (7.1%)	17 (8.3%)	0.004
Late SSI	12 (1.4%)	17 (1.7%)	4 (7.1%)	13 (6.3%)	<0.001

SSI, surgical site infection; TRAM, transverse rectus abdominis muscle.

**Table 2:** Microorganisms responsible for surgical site infection in breast cancer patients who underwent breast surgery at the Department of Surgical Oncology, Medical University of Gdansk in Poland in 2012–2016.

	Classic Breast Surgery (*n* = 865)	Breast-Conserving Surgery (*n* = 1002)	Breast Reconstruction via the TRAM Flap Method (*n* = 56)	Subcutaneous Amputation with Simultaneous Reconstruction (*n* = 206)	*p*-Value
MSSA	22 (47.83%)	16 (33.33%)	3 (37.50%)	11 (36.67%)	0.529
CNS	7 (15.22%)	10 (20.83%)	0 (0.00%)	6 (20.00%)	0.499
*Streptococcus* spp.	3 (6.52%)	4 (8.33%)	1 (12.50%)	1 (3.33%)	0.763
*Enterococcus faecalis*	5 (10.87%)	4 (8.33%)	4 (50.00%)	4 (13.33%)	0.012
*Enterobacteriaceae*	13 (28.26%)	7 (14.58%)	0 (0.00%)	1 (3.33%)	0.016
*Pseudomonas/Acinetobacter*	6 (13.04%)	3 (6.25%)	1 (12.50%)	1 (3.33%)	0.428
Anaerobes	3 (6.52%)	4 (8.33%)	0 (0.00%)	0 (0.00%)	0.370

MSSA, methicillin-susceptible *Staphylococcus aureus*; CNS, coagulase-negative *Staphylococcus*; TRAM, transverse rectus abdominis muscle.

**Table 3:** Antibiotic susceptibility of *Staphylococcus*, *Streptococcus* spp. and Gram-negative bacilli strains isolated from breast cancer patients with surgical site infection at the Department of Surgical Oncology, Medical University of Gdansk in Poland in 2012–2016.

		Strains						
		*Staphylococcus aureus*	Coagulase-negative *Staphylococcus*	*Enterococcus faecalis*	*Streptococcus* spp.	*Enterobacteriaceae*	*Pseudomonas* /*Acinetobacter*	Anaerobes
Number of Strains		52	23	17	9	21	11	7
Antibiotics	PE	0%	0%	100%	100%	---------	---------	100%
	MET	100%	78%	---------	---------	---------	---------	---------
	GE	100%	100%	---------	---------	---------	---------	---------
	GE High	---------	---------	100%	---------	---------	---------	---------
	S High	---------	---------	100%	---------	---------	---------	---------
	TYG	---------	---------	100%	100%	---------	---------	---------
	TEI	---------	---------	100%	100%	---------	---------	---------
	AN	100%	100%	---------	---------	100%	100%	---------
	E	89%	78%	nr	89%	---------	---------	---------
	CC	89%	78%	nr	---------	---------	---------	43%
	SXT	100%	91%	nr	---------	91%	100%	---------
	CIP	98%	91%	87%	100%	96%	100%	---------
	VA	100%	100%	100%	100%	---------	---------	---------
	LZD	100%	100%	100%	100%	---------	---------	---------
	AM	100%	78%	100%	100%	26%	---------	100%
	AMC	100%	78%	100%	100%	26%	---------	100%
	CXM	100%	78%	nr	100%	96%	---------	---------
	CTX	100%	78%	nr	100%	96%	88%	---------
	CAZ	100%	78%	nr	100%	96%	88%	---------
	IPM	100%	78%	100%	100%	100%	100%	---------
	MEM	100%	78%	100%	100%	100%	100%	---------
	MTZ	---------	---------	---------	---------	---------	---------	100%

---------, not applicable/not tested; nr, natural resistance; PE, penicillin; MET, methicillin; GE, gentamicin; AN, amikacin; E, erythromycin; CC, clindamycin; SXT, trimethoprim/sulfamethoxazole; CIP, ciprofloxacin; VA, vancomycin; LZD, linezolid; GE High, high concentration of gentamicin; S High, high concentration of streptomycin; TYG, tigecycline; TEI, teicoplanin; AM, ampicillin; AMC, amoxicillin/clavulanic acid; CXM, cefuroxime; CTX, cefotaxime; CAZ, ceftazidime; IPM, imipenem; MEM, meropenem; MTZ, metronidazole.
